# New approach to prepare cytocompatible 3D scaffolds via the combination of sodium hyaluronate and colloidal particles of conductive polymers

**DOI:** 10.1038/s41598-022-11678-8

**Published:** 2022-05-16

**Authors:** Thanh Huong Truong, Lenka Musilová, Věra Kašpárková, Daniela Jasenská, Petr Ponížil, Antonín Minařík, Eva Korábková, Lukáš Münster, Barbora Hanulíková, Aleš Mráček, Petra Rejmontová, Petr Humpolíček

**Affiliations:** 1 Present Address: Centre of Polymer Systems, Tomas Bata University in Zlin, Zlin, Czech Republic; 2 Faculty of Technology, Tomas Bata University in Zlin, 760 01 Zlin, Czech Republic

**Keywords:** Biomaterials, Biomaterials, Colloids, Gels and hydrogels

## Abstract

Bio-inspired conductive scaffolds composed of sodium hyaluronate containing a colloidal dispersion of water-miscible polyaniline or polypyrrole particles (concentrations of 0.108, 0.054 and 0.036% w/w) were manufactured. For this purpose, either crosslinking with *N*-(3-dimethylaminopropyl-*N*-ethylcarbodiimide hydrochloride and *N*-hydroxysuccinimid or a freeze-thawing process in the presence of poly(vinylalcohol) was used. The scaffolds comprised interconnected pores with prevailing porosity values of ~ 30% and pore sizes enabling the accommodation of cells. A swelling capacity of 92–97% without any sign of disintegration was typical for all samples. The elasticity modulus depended on the composition of the scaffolds, with the highest value of ~ 50 kPa obtained for the sample containing the highest content of polypyrrole particles. The scaffolds did not possess cytotoxicity and allowed cell adhesion and growth on the surface. Using the in vivo*-*mimicking conditions in a bioreactor, cells were also able to grow into the structure of the scaffolds. The technique of scaffold preparation used here thus overcomes the limitations of conductive polymers (e.g. poor solubility in an aqueous environment, and limited miscibility with other hydrophilic polymer matrices) and moreover leads to the preparation of cytocompatible scaffolds with potentially cell-instructive properties, which may be of advantage in the healing of damaged electro-sensitive tissues.

## Introduction

Bioelectrical signals are one of the key stimuli in the development and regeneration of tissues^1^. Due to this fact, electrical stimulation has been studied as a promising tool in disease treatment and wound healing^2^ for decades. In this context, the development of stimuli-responsive electroconductive biomaterials is of great interest when considering the potential use of such materials in a wide variety of biomedical applications^3^. Electroconductivity and stimuli responsivity of such materials can be used as cell instructive properties crucial for the development of modern biomaterials. Indeed, to facilitate electrical stimulation, it is crucial to use electrically conductive materials. Here, different classes of materials can be considered; these include metals, conductive polymers, and piezoelectric materials, which can all transfer electrical, electrochemical, and potentially even electromechanical stimuli to cells^4^. Recently, the preparation of electrically conductive polymers (CP), such as polypyrrole (PPy) and polyaniline (PANI), has become a highly prized goal in the tissue engineering community, as they can efficiently transfer electrical signals at the interfaces with cells due to their intrinsically combined electronic and ionic conductivity^5^. Owing to ionic conductivity, the electrical signal can be transduced to an ionic signal, which is a natural communication system in cells^6^. Consequently, CPs can be highly beneficial in tissue engineering (TE), especially with respect to cardiac, muscle, and nerve tissues^7,8^. Utilizing ionic conductivity can facilitate nerve regeneration in response to electrical stimulation^9,10^. Among additional advantages of CPs is their easy synthesis. Both PANI and PPy can be prepared in various forms such as powders^11^, colloidal dispersions^12^, or films^13^, of which the form facilitating the processing and/or preparation of composites (colloidal dispersions) is especially important, as it can overcome one of the disadvantages of CP, its incompatibility with an aqueous environment. Another shortcoming of CPs is their inability to be prepared in a three-dimensional form (scaffolds). Therefore, naturally derived polymers, such as sodium hyaluronate (HA), have been employed to overcome the limitations related to the fabrication of suitable conductive 3D scaffolds with the required mechanical, viscoelastic, and biological properties^14^.

Recently, many studies have demonstrated that HA plays a crucial role in various biological processes, including cell proliferation, migration, and differentiation, and in the activation of anti-inflammatory growth factors^15,16^. Sodium hyaluronate is a naturally-occurring glycosaminoglycan exhibiting intriguing viscoelastic properties, excellent biocompatibility, biodegradability, and the ability to be easily modified with various functional groups^17^. Moreover, it possesses other benefits for TE, including the absence of toxicity and non-thrombogenic and non-immunogenic properties, in contrast to other synthetic materials^18^. As a constituent of the extracellular matrix, HA is a candidate component for use in the synthesis of scaffolding materials for TE^19^.

The combination of HA with conductive polymers has previously been studied for use in various applications, e.g. for the preparation of theranostic particles loaded with doxorubicin^20^ or hydrogels for TE combining PPy-HA conjugates polymerized with PPy^21^. Collier et al. demonstrated that HA present in HA-doped PPy improved surface morphology and biocompatibility, and significantly promoted vascularization in vivo in comparison with poly(styrenesulfonate)-doped PPy^22^. The molecular weight of HA must be considered as an essential parameter, as it plays an important role in the hydrophilicity and cell receptor signalling of HA-based materials^23^. Kim et al. reported that the behaviour of PPy-HA-based biomaterials differed according to the molecular weight (MW) of HA (in the range 35 × 10^3^–2 × 10^6^ Da), with PPy/high-MW HA exhibiting higher electrochemical activity, lower impedance, and higher hydrophilicity. Meanwhile, PPy/lower-MW HA supported the adhesion and growth of fibroblasts and neuronal cells, while high-MW HA-doped PPy resisted cell growth due to the higher hydrophilicity of its surface^21^. According to Shin et al., HA hydrogel significantly promoted the neuronal differentiation of human fetal neural stem cells and human-induced pluripotent stem cell-derived neural progenitor cells^24^. Texidó et al. reported the modification of PPy with dopaminated hyaluronic acid, which improved adhesion onto a poly(dimethylsiloxane) substrate, thus forming a flexible composite film. The film demonstrated enhanced water resistance and electrical conductivity under mechanical stimuli compared with bare PPy films and PPy with undopaminated hyaluronic acid^25^. So far only a limited number of studies have dealt with the use of PANI and HA for the fabrication of electrically conductive scaffolds. One recent study dealing with this topic highlighted the induction of cardiomyocyte proliferation within lamellar scaffolds based on a colloidal complex of water-miscible PANI with collagen, fibroin, and hyaluronate^26^. In scientific literature various studies on HA-PPy composites suitable for the preparation of scaffolds have already been published. Recently the use of cryogel- or hydrogel-based scaffolds as well as 3D printed structures has become increasingly common. In the field of conductive polymers, cryogels allow the combination of PANI or PPy with synthetic polymers, e.g. poly(vinyl alcohol)^27,28^. The main problem with cryogels is related to their small pore size and internal architecture limiting cell ingrowth. Their cytocompatibility is, however, adequate. An interesting study by Lu et al. described the preparation of PPy hydrogels by means of oxidative polymerization with ammonium peroxysulfate^29^. Unfortunately, no cytocompatibility studies were provided for these materials and, thus, it is difficult to evaluate the applicability of this concept to TE. The preparation of pyrrole-HA-conjugates showed good cytocompatibility; however, cell ingrowth was not studied in this case either^30^. Currently, hand in hand with the development of so-called additive technologies, which are suitable for the preparation of 3D-structures, printable materials containing CP are being developed.

As far as the authors know, no previously published study has investigated scaffolds formed by the here presented technique, these prepared by the chemical and physical crosslinking of the biopolymer sodium hyaluronate (HA) with incorporated CP-based colloidal particles. In this work, such scaffolds were prepared using embedded conductive and water-soluble colloidal particles of PPy or PANI also stabilized with HA. This approach has at least two advantages with respect to scaffolds containing conductive polymers, namely the resulting presence of CP miscibility with water and the ability to prepare scaffolds containing cell-instructive biopolymers. The main aim of the study was to investigate whether—and if so, how—the method of crosslinking and the composition of the material influence the physico-chemical and biological properties of the scaffolds in terms of cytocompatibility and the ability to facilitate the ingrowth of cells. The new knowledge obtained here on the impact of crosslinking technique on the resulting materials could be applicable to additive technologies, which could employ the composite materials developed here. In sum, this original approach was investigated with the aim of preparing porous, conductive, and cytocompatible scaffolds for TE.

## Materials and methods

### Materials

Reagent-grade aniline hydrochloride (≥ 98%), pyrrole (98%), ammonium peroxydisulfate (98%) and poly(vinyl alcohol) (PVA; molecular weight 40 × 10^3^ g mol^−1^) were purchased from Sigma Aldrich (Germany) as well as the crosslinking agents, *N*-(3-dimethylaminopropyl-*N*-ethylcarbodiimide hydrochloride (EDC) and *N*-hydroxysuccinimid (NHS). Sodium hyaluronate (HA; molecular weight 1.8–2.1 × 10^6^ g mol^−1^) was a kind gift from Contipro a.s. (Czech Republic).

### Synthesis of polyaniline and polypyrrole colloids

Samples of colloidal PANI and PPy were prepared via the oxidation of the respective monomer, aniline hydrochloride (AH) or pyrrole (Py), with ammonium peroxydisulfate (APS) in the presence of sodium hyaluronate (HA). The colloidal particles are designated HaPANI and HaPPy. Briefly, HA was dissolved to a 1% (w/w) solution in MilliQ water under stirring at 55 °C overnight. For dispersion polymerization, AH (0.2 M) in 1% (w/w) HA solution was prepared, and polymerization was started at room temperature (20 ± 2 °C) by adding 0.1 M aqueous APS solution to the reaction mixture. The mixture was stirred for 5 min, and left at rest to polymerize. The reaction was completed within 2 h. Correspondingly, PPy was prepared using 0.2 M Py monomer solution in 1% (w/w) HA and 0.25 M aqueous APS, which were mixed and left to polymerize for 2 h. The composite character of both samples proving the presence of both conductive polymer and HA was confirmed by FTIR analysis. For HaPANI it is given in^13^, and the spectrum of the HaPPy is shown in Fig. S4.

In order to remove residual impurities, the colloidal dispersions were transferred into Spectra Por 2 membrane dialysis tubing (cut-off 12,000–14,000; Spectrum Laboratories Inc., U.S.) and purified through exhaustive dialysis against 0.2 M hydrochloric acid. Water was not used as a dialysis medium in order to avoid the gradual deprotonation of HaPANI and HaPPy conductive particles to their non-conductive counterparts in a neutral environment. Each of the colloidal dispersions was employed for the preparation of scaffolds.

### Preparation of scaffolds

Samples were prepared in two different ways, specifically by means of chemical (^Ch^) and physical (^Ph^) crosslinking procedures. The composition of the samples is given in Table 1.

**Table 1:** Composition of scaffolds.

Sample	Composition (w/w)						
	HA	PVA	PANI	PPy	NHS		EDC
HaPANI^Ch_108^	1	×	0.108	×	0.013		0.013
HaPANI^Ch_54^	1	×	0.054	×	0.013		0.013
HaPANI^Ch_36^	1	×	0.036	×	0.013		0.013
HaPPy^Ch_108^	1	×	×	0.108	0.013		0.013
HaPPy^Ch_54^	1	×	×	0.054	0.013		0.013
HaPPy^Ch_36^	1	×	×	0.036	0.013		0.013
HaPANI^Ph_108^	1	0.5	0.108	×	×		×
HaPANI^Ph_54^	1	0.5	0.054	×	×		×
HaPANI^Ph_36^	1	0.5	0.036	×	×		×
HaPPy^Ph_108^	1	0.5	×	0.108	×		×
HaPPy^Ph_54^	1	0.5	×	0.054	×		×
HaPPy^Ph_36^	1	0.5	×	0.036	×		×

The samples are labelled according to the type of CP-based colloidal particles (PANI, PPy); the method of crosslinking, i.e. chemical (Ch) or physical (Ph); and the concentration of CP-based colloidal particles.

On the basis of preliminary studies, hyaluronan scaffolds containing PANI or PPy colloids chemically crosslinked with EDC and NHS (designated HaPANI^Ch^, HaPPy^Ch^) were made according to Gřundělová et al.^31^. A stock solution of HA with a concentration of 1% (w/w) was prepared by dissolving the polymer in Milli-Q water for 24 h at 50 °C. The crosslinking agents EDC (100 mM) and NHS were added to the 1% (w/w) HA solution in a ratio of 1:1.5 (w/w) and the mixture was stirred for 1 h at 25 °C. Subsequently, the solution was titrated under continuous stirring, with PANI colloid dispersed in 0.2 M HCl to adjust the pH to 4.5–4.7. Correspondingly, PPy-containing scaffolds were prepared.

The crosslinking of scaffolds using the physical freeze–thaw method^32–34^ was carried out using PVA, and the samples were designated HaPANI^Ph^, HaPPy^Ph^. A stock solution of PVA with a concentration of 10% (w/w) was prepared by dissolving the polymer in Milli-Q water for 24 h at 80 °C. The PVA was added to the 1% (w/w) HA solution in the ratio 1:2 (w/w) and stirred for 1 h at 25 °C. Subsequently, the solution was titrated under continuous stirring, with the colloidal dispersion of PANI (in 0.2 M HCl) to adjust the pH to 4.5–4.7. Correspondingly, PPy-containing scaffolds were prepared. The concentrations of CP-based colloidal particles in the scaffolds were 0.108, 0.054 and 0.036% (w/w), these values used as superscripts for sample labelling.

The solutions from both preparation routes (chemical or physical) were then poured into polystyrene moulds and first frozen at − 18 °C for 60 h before being freeze-dried in an ALPHA1-2 LD plus freeze-dryer (Christ, UK). As a result, cylindrical specimens were obtained with a diameter of 28 mm and a height of 5 mm.

### Physico-chemical characterization

#### Size and morphology of colloidal particles

The particle size and size distribution were determined on dialysed samples using dynamic light scattering (DLS) (Zetasizer Nano ZS, Malvern Instrument, UK). The hydrodynamic radii of colloidal particles, expressed as z-average particle diameters, were measured at 25 °C at a scattering angle of 173°. The polydispersity index (PDI) describing the width of the particle size distribution in a given sample was also determined. Samples were prepared for measurement by diluting 10 μL of freshly prepared dispersion in 1 mL of 0.1 M HCl. The analyses were run in triplicates with average values and standard deviations reported.

The morphology of colloidal particles was assessed with a JEOL JEM 2000 FX transmission electron microscope (TEM) (Japan). Scanning electron microscopy (SEM) analysis was carried out using Nova NanoSEM 450 (FEI, Czech Republic) microscope operated at acceleration voltages of 5 kV (magnification up to 300 000x) and 10 kV (magnification 500 000x).

#### Mechanical properties

Mechanical properties in terms of Young’s modulus were determined on an Autograph AG–X tensile tester (Shimadzu). The analyses were performed on cylindrical samples with a length of 12 mm and a diameter of 8 mm. Each sample (Fig. S1) was inserted between two parallel plates and deformed at a rate of 1 mm min^−1^. The measurement time was 3 min and within this short period of time the humidity of the sample was considered constant. Young’s modulus was determined as the slope of the linear part of the stress–strain curve. The experiments were performed in compression mode and the relative standard deviation of all values obtained did not exceed 5%.

#### Surface topography and electrical properties

The surface electrical properties of the scaffolds were analysed by tunneling atomic force microscopy (AFM) using the PeakForce TUNA module (Fig. S2) on a Dimension ICON instrument (Bruker Corporation; US). The TUNA current is the average current over one full tapping cycle in PeakForce mode. Peak current corresponds to the current measured at a defined maximum force in the PeakForce cycle. Measurements were performed at ambient temperature using a PFTUNA probe (Bruker Corporation; US) with a spring constant of 0.4 N m^−1^, covered on both sides with a conductive Pt/Ir layer. The scanning rate was 0.3 Hz and the TUNA and peak currents were measured by applying a bias voltage of 10 V. The resulting parameters of area roughness and TUNA and peak currents were determined according to the protocol of ISO standard 25178–2 using NanoScope Analysis software v. 1.5. The following parameters were determined: surface roughness (*S*_*a*_), maximal surface height (*S*_*z*_), average and maximum TUNA current, and average and maximum peak current. Data from AFM were processed using Gwyddion 2.5 software (Czech Metrology Institute, Czech Republic). To ensure constant characterization conditions, a copper tape with acrylic conductive adhesive (ELCHEMOCo, Prague, Czech Republic) was situated onto the scaffold surface to a distance about 150 μm from the tip of the AFM probe, see supplementary data.

#### Morphology and porosity of scaffolds

The surface morphology of lyophilized samples was studied using a scanning electron microscope (Phenom Pro desktop scanning electron microscope (SEM) with a BSE detector (Phenom-World B.V., The Netherlands)) at acceleration voltages of 10 and 15 kV. The samples (Fig. S3) were cut into sections using a sharp razor blade, to observe the cross-sectional morphologies of their scaffolds. The pore sizes of the scaffolds were measured by analysing the SEM images captured, as described above. The method of image analysis was based on the chord length method used in the ASTM standard ASTM E-112 (E-112, 2010). A system of intersecting lines was drawn on the SEM images of the planar sections of the samples, and chord lengths were automatically measured by an in-house algorithm written in the Python programming language. Then, the mean pore size was computed using the protocol of ASTM E-112 standard and expressed as a percentage of pore volume relative to the total volume of the sample.

#### Swelling of scaffolds

The swelling degree (*SD*) of the lyophilized samples was determined gravimetrically. Weighted, disk-shaped specimens were immersed in phosphate buffered saline (PBS) at 37 °C to reach swelling equilibrium. The water uptake was determined by removing the swelled samples from the PBS at selected time intervals, wiping them with tissue paper, and weighing them. The *SD* was calculated according to the equation *S*_*D*_ = (*W*_*S*_ − *W*_*D*_)/*W*_*D*_ × 100 (%), where *W*_*S*_ and *W*_*D*_ are the weights of the swollen and dry sample, respectively. The presented results are average values from four independent measurements reported with standard deviations.

### Cytocompatibility of scaffolds

The cytocompatibility of the scaffolds was determined using the ATCC CRL-1658 NIH/3T3 mouse embryonic fibroblast cell line. ATCC-formulated Dulbecco's Modified Eagle's Medium, catalogue no. 30-2002, containing 10% calf serum (BioSera, France) and 100 U mL^−1^ penicillin/streptomycin (GE Healthcare HyClone, UK) was used as the cultivation medium in all experiments.

The cytocompatibility testing consists of following steps: determination of cytotoxicity according to ISO standard → confirmation of ability of cells to adhere and proliferate on the surface of scaffold under static cultivation condition → testing of ability of cells to grow into the structure of scaffold under in vivo mimicking conditions using bioreactor.

#### Cytotoxicity

The cytotoxicity was investigated according to ISO protocol 10993–5, both on freshly prepared scaffolds and after their lyophilization. For the test, 0.1 g of tested material was extracted with 1 mL of cultivation medium for 24 h at 37 °C under stirring. The cells were pre-cultivated for 24 h and seeded at a concentration of 1 × 10^5^ per well (96 well plates were used). The extracts were diluted with culture medium to obtain a series of dilutions with concentrations of 75, 50, 25, 20, 10, and 1%. After incubation for 24 h, the cell viability was determined by 3-(4,5-dimethylthiazol-2-yl)-diphenyltetrazolium assay (MTT cell proliferation assay kit, Duchefa Biochemie, Netherlands). The absorbance was measured at a wavelength of 570 nm using an Infinite m200pro luminometer (Tecan, Switzerland). The viability of the cells was expressed as the reduction in cell viability relative to the reference, i.e., cells cultivated in the absence of extracts. All tests were conducted in quadruplicates. The morphology of the cells was observed using an inverted Olympus phase contrast microscope (Olympus IX81, Japan).

#### Proliferation test

The ability of cells to proliferate on the tested samples after their lyophilisation was determined using the ATCC CRL-1658 NIH/3T3 mouse embryonic fibroblast cell line. A cell suspension at a concentration of 1 × 10^6^ cells per mL was prepared, and 0.3 mL of the suspension was carefully injected by syringe into each scaffold and onto its surface, after which the scaffolds were placed into an incubator for 2 h to induce cell adhesion and initiate proliferation. After this period, cultivation medium was poured over the scaffold, and the scaffold was cultivated for 7 days. The medium was changed every second day of the experiment. After the cultivation period, the cells were fixed and stained with Hoechst 33,258 (Molecular Probes, Carlsbad, CA, USA) and ActinRed 555, which binds selectively with F-actin (Life Technologies, USA). The morphology of the cells was investigated using a confocal microscope (Olympus Fluoview FV 3000, Japan).

#### Cell ingrowth into scaffolds

Before testing the scaffolds in the 3D environment of a bioreactor, cells were seeded onto the scaffolds and pre-cultivated for 24 h under the standard conditions given above. After pre-cultivation, the samples were placed into a bioreactor, where they were cultivated for the next 7 days to allow cell ingrowth. For testing, an RCCS-4 rotary cell culture system (Synthecon Incorporated, USA) was used. Each of the samples was separately inserted inside the bioreactor and 50 mL of cultivation medium was added. The forward rotation of the reactor was adjusted to 10.5 rpm. The partial oxygen pressure, as well as the temperature were the same as under standard cultivation in an incubator. After cultivation, the scaffolds were fixed with 4% paraformaldehyde overnight, washed with PBS, permeabilized with 0.5% Triton X-100 (Sigma Aldrich, Germany), and washed again three times with PBS. The cells were stained using ActinRed™ 555 (Thermo Fisher Scientific, USA). The tested samples were sliced, and the cell morphology was observed using a confocal microscope (Olympus Fluoview FV 3000, Japan). Images were taken from the cross-sections of scaffolds, approximately 0.5 cm below the surface. The images thus represent cells which grow into the structures of scaffolds from their surfaces.

## Results and discussion

### Conductive colloidal dispersions

Colloidal dispersions of CP stabilized with polysaccharides are smart formulations helping to solve the problem of the poor solubility of CP in aqueous environment, and which improve the cytocompatibility of the final composite^35^. Another, not previously mentioned advantage of such composites appears in the case when CPs are mixed with other hydrophilic polymer matrices—for example, when polymer-based scaffolds are fabricated. Here, the better polymer–polymer compatibility of HaPANI or HaPPy colloids containing HA on one side combined with the bulk polymer of the scaffold on the other side (also based on HA) is expected in comparison with pristine PANI or PPy.

**Figure 1:**
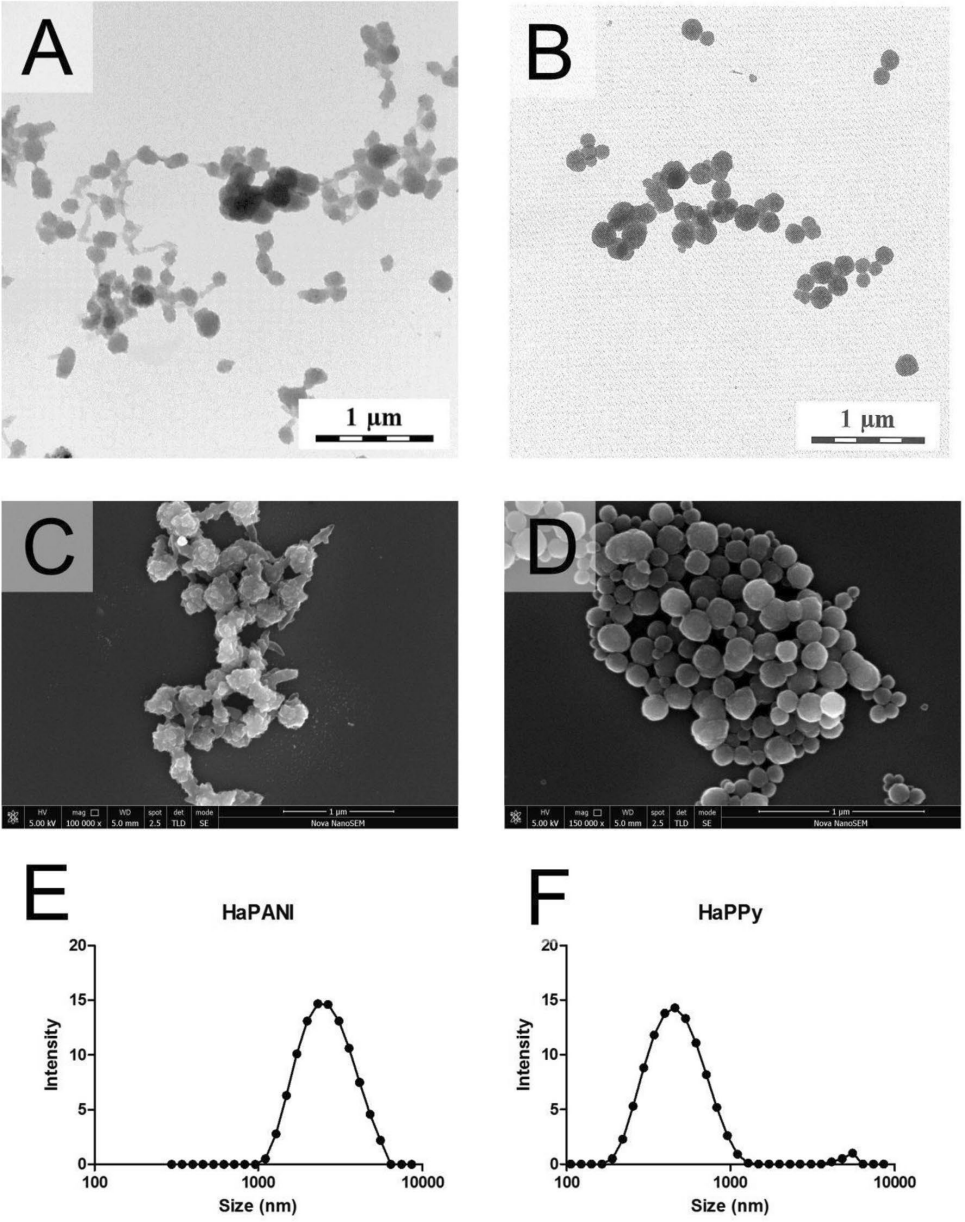
Transmission electron micrographs of CP-based colloidal particles stabilized with sodium hyaluronate HaPANI (**A**), HaPPy (**B**); scanning electron micrographs of corresponding particles HaPANI (**C**), HaPPy (**D**) and particle size distribution curves of HaPANI (**E**), HaPPy (**F**). The particle size analyses were run in triplicates.

The size and polydispersity index of colloidal particles used for scaffold fabrication were determined on thoroughly dialysed samples, purified of possible impurities (e.g. monomers, residual precursors, crosslinkers etc.) which might cause cytotoxic effects^36^. The hydrodynamic diameters of particles were different, with micrometer sizes observed for HaPANI (2200 ± 260 nm) and smaller sizes determined for HaPPy (447 ± 8 nm). Both samples showed, however, similar polydispersity indexes (~ 0.2). The morphology of particles observed by transmission electron microscopy (Fig. 1A,B) conformed with scattering measurements (Fig. 1E,F), showing in both cases regular, spherical particles with a smooth surface, and, in the case of HaPANI, the interconnection of such particles to form bigger, loose clusters. As for presence of the clusters, appearance of HaPANI sample captured by SEM complies, in fact, with TEM visualisation. Particles in Fig. 1C show irregular, bumpy surface with small protrusions visible on each unit. The particles are joined by rods/fibres which are also covered with the protrusions and which may originate from the stabilizing hyaluronate chains. The sizes of primary particles are smaller than their average diameter determined by DLS, which can be caused by clustering of HaPANI and obviously from different measuring principle of DLS and microscopy techniques. The different surface morphology was, however, observed for the HaPPy colloid in Fig. 1D, which contained round and smooth particles.

The stability of the colloids was determined after two months storage at temperature of 5 ± 2 °C by measuring their particle sizes. In case of Ha-PPy, the particle size was reduced by storage to 87% of the original value. The similar size decrease was reported for PVA stabilized PPy colloid after 3 months storage, which was attributed to the disentanglement of some colloidal aggregates developed immediately after polymerization^37^. Particle size reduction after six months of storage was also observed for chitosan-stabilized PANI colloids by Kasparkova et al.^35^. On the contrary, the same authors reported an increase in the size for PANI stabilized with high-molecular-weight hyaluronate (MW 1.8–2.1 × 10^6^, 6 months storage). This increase corresponds with the results of the stability test in this work, where the size of HaPANI particles grew to 113% of the original value after two months storage. The obtained data illustrate that changes in particle sizes of composite colloids depend on the composition of the sample, in particular on the type of conductive polymer present in the colloids. However, it should be emphasized that the scaffolds were fabricated with the newly-prepared colloidal particles.

### Preparation and characterization of scaffolds

The CP-based colloidal dispersions HaPANI and HaPPy were used, each incorporated into the used HA matrix and in three different concentrations. Two methods of scaffold preparation were employed, each followed by an identical freeze-drying procedure. The first route included chemical crosslinking with EDC and NHS, the latter used as the activator of carboxylic groups; the second procedure involved crosslinking by the freeze–thaw method using PVA, hereinafter referred to as the physical route. As far as the authors know, no previously published study has investigated the here presented technique, thus the question, hence, arose whether—and if so, how—these two preparation methods together with scaffold composition affect the material and biological properties of the given scaffolds.

### Mechanical properties

**Figure 2:**
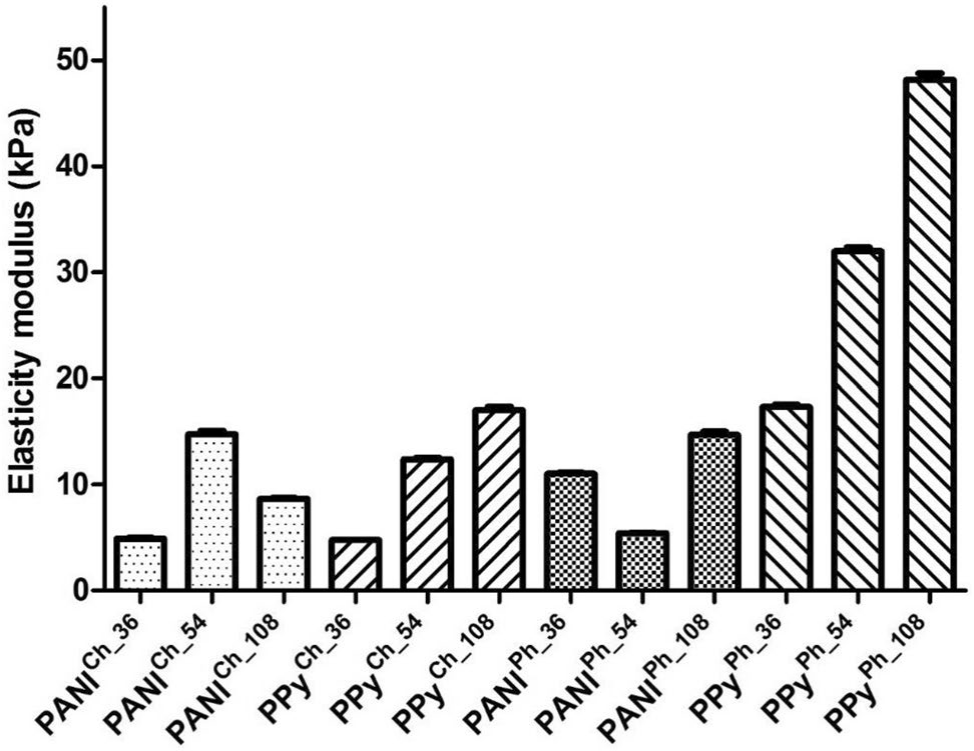
Elasticity moduli calculated for physically (^PH^) and chemically (^CH^) crosslinked scaffolds. The experiments were performed in compression mode and the relative standard deviation of all values obtained did not exceed 5%.

Mechanical properties defined in terms of the modulus of elasticity (*E)* for samples of HaPPy^Ph^ crosslinked by freeze-thawing in the presence of PVA were significantly different to those for chemically crosslinked HaPPy^Ch^ and scaffolds containing HaPANI colloidal particles (Fig. 2). Their *E* values were the highest (17–50 kPa) and strongly depended on the content of PPy particles in the samples, with the highest value observed for HaPPy^Ph_108^. A corresponding dependence of the elasticity modulus on the content of colloidal particles was seen for chemically crosslinked HaPPy^Ch^, though with significantly lower *E* of only 5–17 kPa. This indicates a stronger network for the samples containing PVA and good mutual compatibility between all the involved components, HA, PVA, and CP-based colloidal particles, of which the last component contributed to the good level of compatibility through the acidic nature of the particle dispersion^38^. Differences between physically and chemically crosslinked samples containing HaPANI (Fig. 2) were only minor in comparison with the corresponding scaffolds prepared with HaPPy. The modulus for physically crosslinked HaPANI^Ph^ ranged from 5 to 15 kPa, with the lowest value observed for the sample with 0.054% (w/w) colloid (HaPANI^Ph_54^). This *E* range was actually similar to the *E* range observed for chemically crosslinked samples (HaPANI^Ch^), i.e., from 5 to 15 kPa; however, the correlation between the *E* values and the amount of colloidal particles is different in these samples. In summary, the scaffolds incorporating HaPANI colloidal particles exhibited a low elasticity modulus, which moreover lacked correlation with the colloid content in the sample. In contrast, the behaviour of all scaffolds containing HaPPy colloid was more predictable in terms of *E *versus particle content. This is surprising, as one would expect more similar behaviour among scaffolds containing PVA irrespective of the type of colloidal particles used, as PVA contributes to a rubbery and elastic texture and a higher mechanical strength of scaffolds thanks to the better distribution of the mechanical load along the crystallites of the three-dimensional scaffold structure^39^. The mechanical properties of such scaffolds hence result from the crosslinking routes and the content/type of colloidal particles used for their fabrication.

### Surface topography and electrical properties

**Figure 3:**
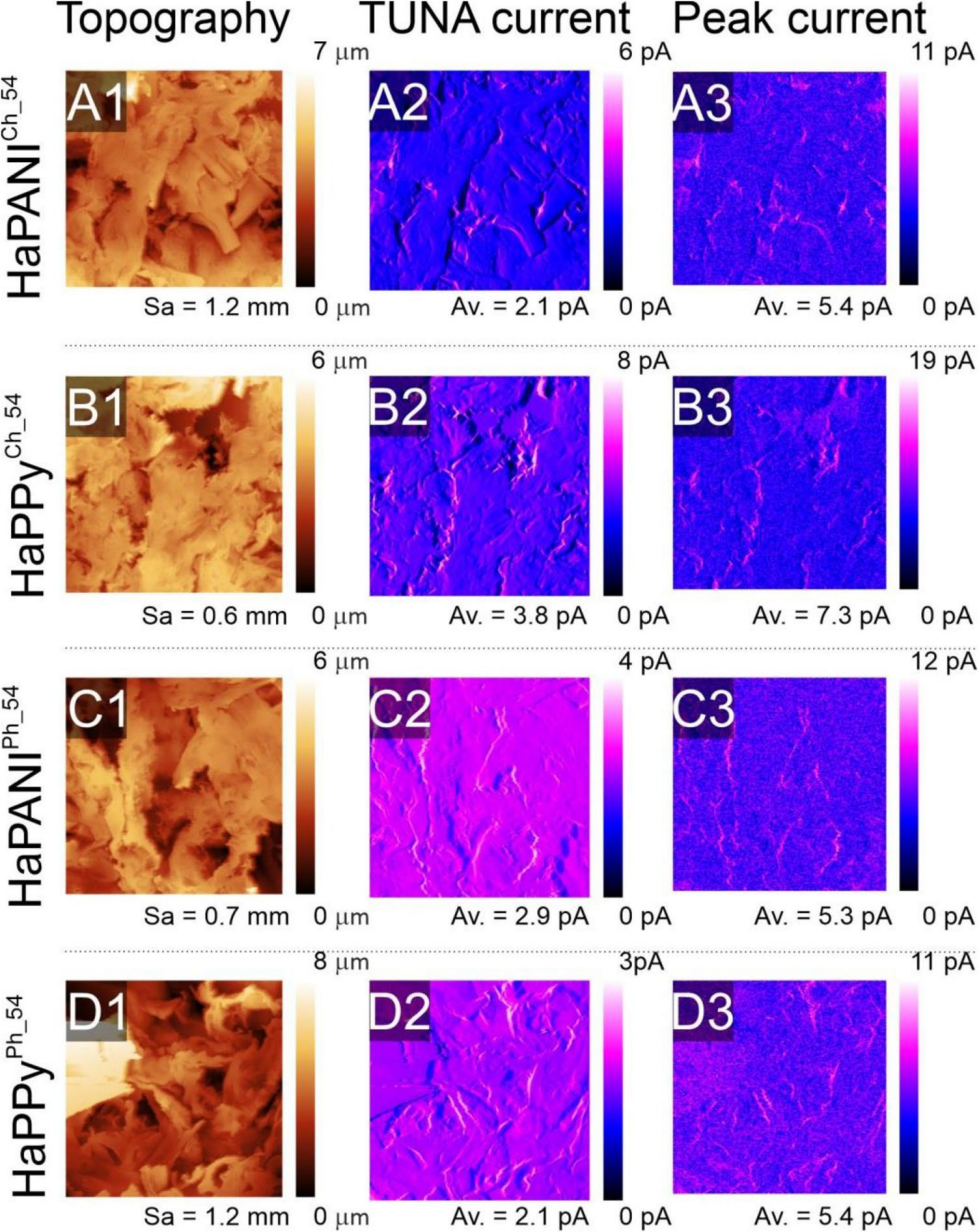
AFM images of HaPANI^Ch^ (**A**), HaPPy^Ch^ (**B**), HaPANI^Ph^ (**C**), HaPPy^Ph^ (**D**). The topography (1), TUNA current (2), and peak current (3) are presented for every sample.

Lyophilized samples with a medium concentration of the conductive component (0.054% w/w) were analysed using AFM to determine their surface properties and surface conductivity (Fig. 3).

The surface roughness (*Sa*) and maximum surface height (*Sz*) of the samples, as presented in Fig. 3, show that the surface characteristics of the scaffolds are rather similar. Their surfaces are irregular with embedded pores, these seen mainly in physically crosslinked samples. Chemical crosslinking gave rise to scaffolds with a flake-like surface structure. The second and third columns of Fig. 3 show current maps expressed as TUNA and Peak currents. These unambiguously prove that the scaffolds are conductive and that their average values of TUNA current are similar, lying in the range of 2.1–3.8 pA, irrespective of the type of conductive particles used and of the fact that the percentage of the conductive component in the chemically crosslinked samples was higher with respect to the total mass of the scaffold than in the scaffolds crosslinked physically. A bigger difference in the measured values can be seen for maximum currents (max TUNA, max Peak current), which were lower for physically crosslinked samples, with a maximum current of about 12 pA. In the case of samples crosslinked chemically, values of 15 and 19 pA for HaPANI^Ch _54^ and HaPPy^Ch _54^, respectively, were measured. This minor difference is a natural consequence of the higher ratio of CP-based colloids in the chemically crosslinked samples. In this respect, both HaPANI and HaPPy colloids performed similarly within the same type of crosslinking mechanism. Certainly, as such samples contain particles of intrinsically conductive polymer (CP) with mixed ionic and electron conductivity, and as the surroundings within hydrated scaffolds are also ionically conductive, the scaffolds thus form a suitable environment which can facilitate the adhesion and proliferation of electrically conductive cells and tissues^24^.

### Porosity and morphology

**Figure 4:**
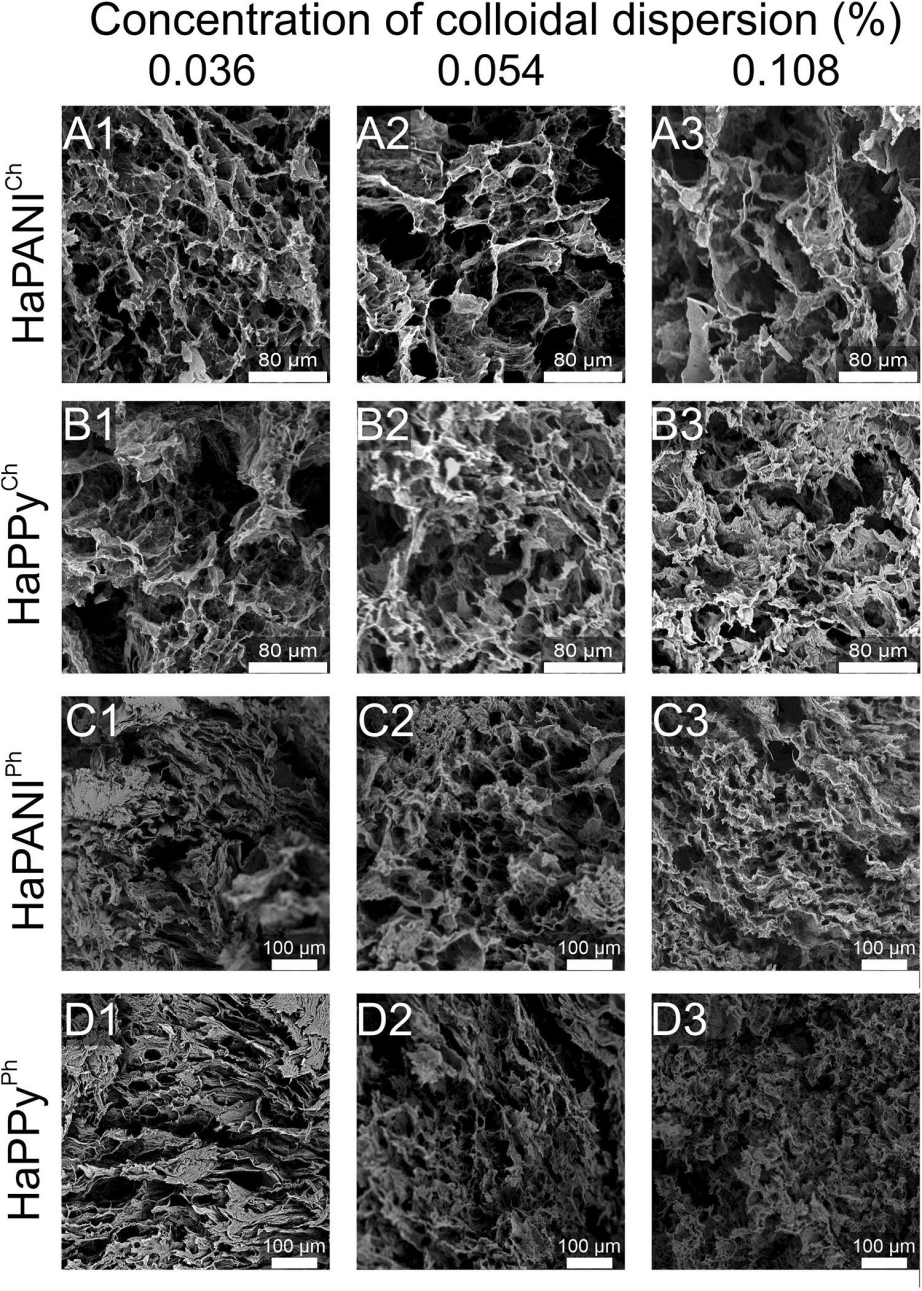
SEM photomicrographs of lyophilized HaPANI^Ch^ (**A**), HaPPy^Ch^ (**B**), HaPANI^Ph^ (**C**) and HaPPy^Ph^ (**D**) containing colloid concentrations of 0.036 (1), 0.054 (2), or 0.108(3) %.

**Figure 5:**
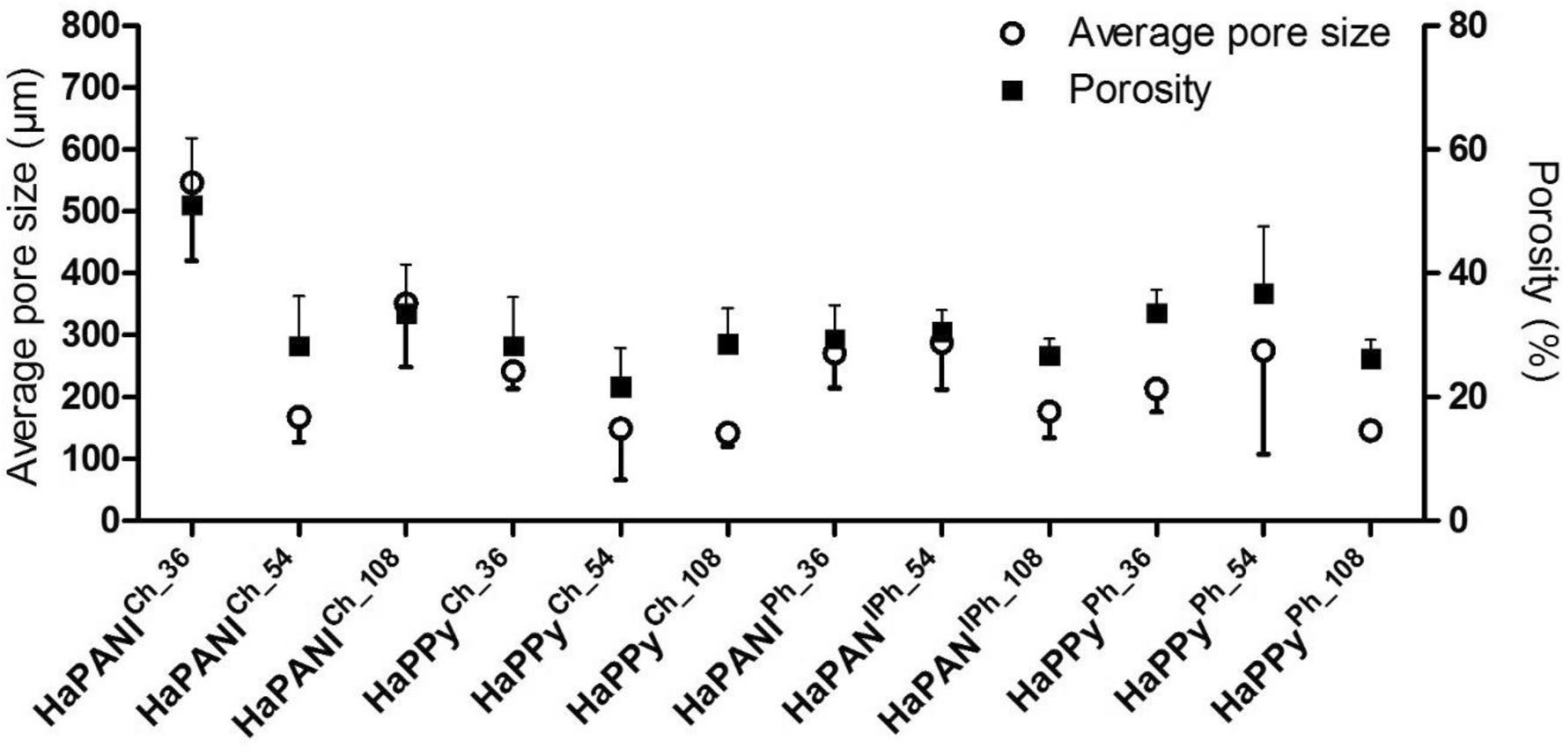
Average pore size and its standard deviation and average porosity and its standard deviation of the scaffolds as determined by image analysis according to ASTM E-112.

Morphology, including the size of pores in lyophilized scaffolds, was assessed by means of the visual observation of SEM images (Fig. 4). The images were used for the measurements of pore size and the calculation of porosity, which were both quantified according to the chord length method given by the protocol of ASTM E-112 (Fig. 5).

According to the results of the image analysis, the physically crosslinked HaPPy^Ph^ samples with the two lower concentrations of colloidal particles (0.036, 0.054%) exhibited the biggest pores in the scaffolds. The pore sizes were the smallest in the sample with the colloid concentration of 0.108% (HaPPy^Ph_108^). The formation of interconnected pores with appropriate diameter is especially important as some other available techniques of scaffold preparation do not provide this. For example the formation of cryogels do not lead to scaffolds with appropriate pores size^27,28^.

In numerical values, the porosity ranged from 26 to 37%, with the lowest value for the highest content of colloidal particles, as observed for HaPPy^Ph_108^. The HaPPy^Ch^ samples, prepared with chemical crosslinking, yielded a lower porosity of 22 to 28%, which was lowest for the scaffold with the medium content of colloidal particles (HaPPy^Ch_54^). The physically crosslinked HaPANI^Ph^ samples were, with respect to pore sizes, similar to the above-discussed samples containing PPy colloidal particles, and showed pore sizes of 271, 288, and 177 µm for scaffolds with increasing concentrations of CP-based colloid. Calculated porosity values for these formulations ranged between 27 and 32%. HaPANI^Ch^ scaffolds, prepared by chemical crosslinking, exhibited porosity values comparable to those of scaffolds crosslinked physically, with one exception: the sample HaPANI^Ch_36^. This outlying sample contained the lowest concentration of HaPANI colloidal particles and exhibited a porosity of more than 50% and an average pore size of 547 ± 126 µm. The porosities of the other two HaPANI-based samples were 30% and 35% for the medium and highest colloid contents, respectively. According to the image analysis supported by visual evaluation, the bigger pores were always seen in samples with the lowest content of colloidal particles and, in general, the sizes of pores in most of the scaffolds were roughly estimated to be within the range of 200–300 µm, which is sufficient for the ingrowth of cells into the structure and for nutrient supply and metabolite removal.

### Swelling

**Figure 6:**
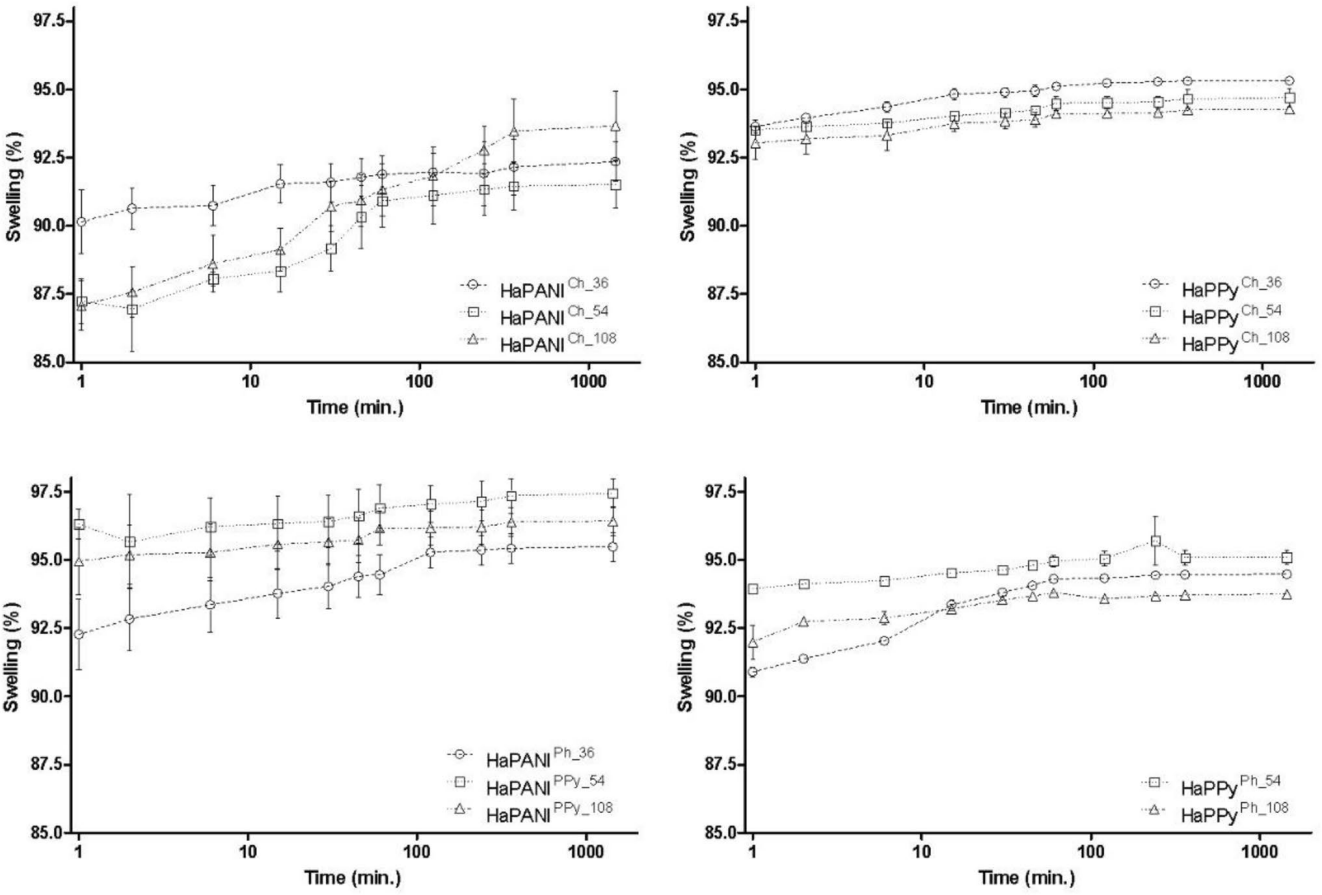
Swelling behaviour, expressed as average swelling degree. The average values from four measurements reported with standard deviations.

The course of swelling was similar for all samples, and the initial increase in swelling degree (*SD*), observed within the first approx. 100 min, was followed by equilibrium (Fig. 6). At equilibrium, the *SD* of physically crosslinked HaPPy^Ph^ scaffolds ranged from 92 to 96% and decreased with growing colloid content in the sample. In comparison, differences in the swelling of all chemically crosslinked HaPPy^Ch^ samples were only minor and their equilibrium *SD* was 93–95%. Scaffolds containing HaPANI exhibited lower *SD* for physically crosslinked samples in comparison with samples that were crosslinked chemically. With short swelling times, the lowest *SD* of 87% was recorded for HaPANI^Ph_54^, while the highest equilibrium *SD* of 93% was observed for the sample containing the highest concentration of particles. Chemically crosslinked HaPANI^Ch^ scaffolds swelled better, their *SD* ranging from 95 to 97%. In this respect, *SD* is influenced by PVA in the hydrogel matrix; in chemically crosslinked samples with PVA absent, the naturally high ability of HA to take up water prevailed and the *SD* was, therefore, higher. When in a mixture, the crystallinity of PVA affects the hydrogel structure, which is less loose compared with samples containing solely HA. As a result, physically crosslinked gels absorb less water, this resulting in lower *SD*. Nevertheless, regardless of the observed differences, the *SD* values were similar and all scaffolds showed good swelling characteristics.

### Cytotoxicity

The absence of cytotoxicity is one of the fundamental requirements that biomaterials must meet. The cytotoxicity of a full set of samples was therefore tested. To obtain representative information, the protocol of ISO standard 10993–5 was employed, including the use of an appropriate cell line; i.e., NIH/3T3 fibroblasts. The cytotoxicity was determined on native samples prior to lyophilisation and on lyophilised scaffolds, which are marked with the superscript ^L^.

**Figure 7:**
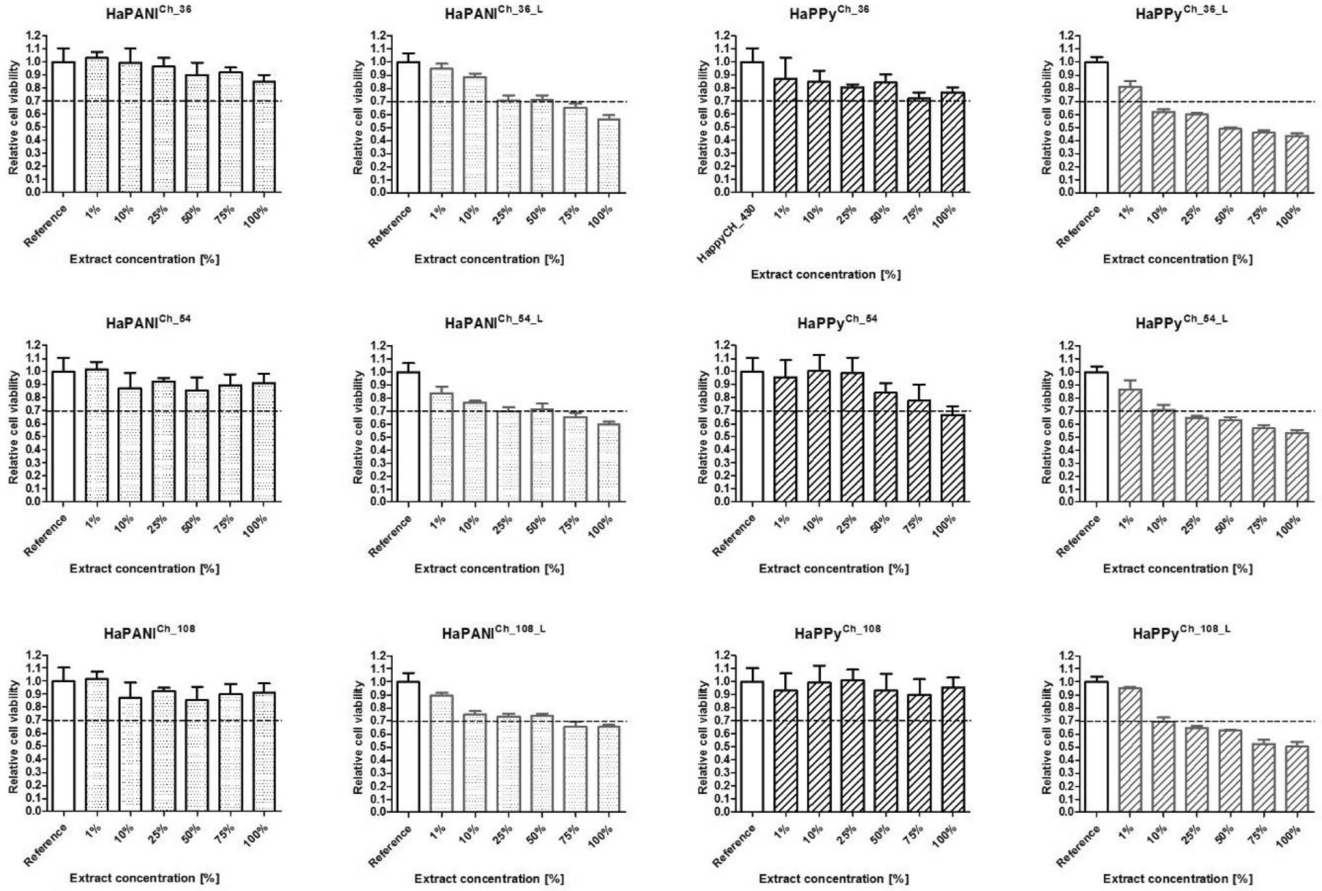
The cytotoxicity of chemically crosslinked scaffolds. According to ISO standard 10993, a decrease in viability to below 70% of the reference corresponds to a cytotoxic effect on the part of the tested materials. Here, the dashed line shows the threshold of cytotoxicity. All tests were conducted in quadruplicates within three repetitions. The average viability with its standard deviation are shown.

The summary of cytotoxicity data recorded for chemically crosslinked scaffolds and presented in Fig. 7 unambiguously demonstrates that all the native scaffolds prepared by chemical crosslinking, HaPANI^Ch^ or HaPPy^Ch^, can be classified as non-cytotoxic, with cell viabilities higher than 70%. The situation was, however, different for lyophilised scaffolds. After lyophilisation, the cytotoxicity increased, especially in the case of HaPPy-containing scaffolds, the lyophilised HaPPy^Ch_L^ scaffolds showing cytotoxic effects with concentrations of extract higher than 25%, irrespective of the concentration of the colloidal particles used. In the case of HaPANI^Ch_L^ containing HaPANI colloid, the cytotoxicity was lower and only the two highest concentrations of extracts (70 and 100%) exceeded the threshold for cytotoxicity, and moreover only slightly. The effect of lyophilisation on cytotoxicity can be related to the protocol of testing given by the ISO standard. The procedure defines the ratio of material/extraction medium according to mass. In the case of lyophilised samples with water removed, the ratio of the mass of dry matter (which, in fact, contains impurities) to the amount of extraction medium is higher. This situation can lead to the higher cytotoxicity of lyophilized samples, despite the fact that the procedure itself should not produce additional cytotoxic compounds.

**Figure 8:**
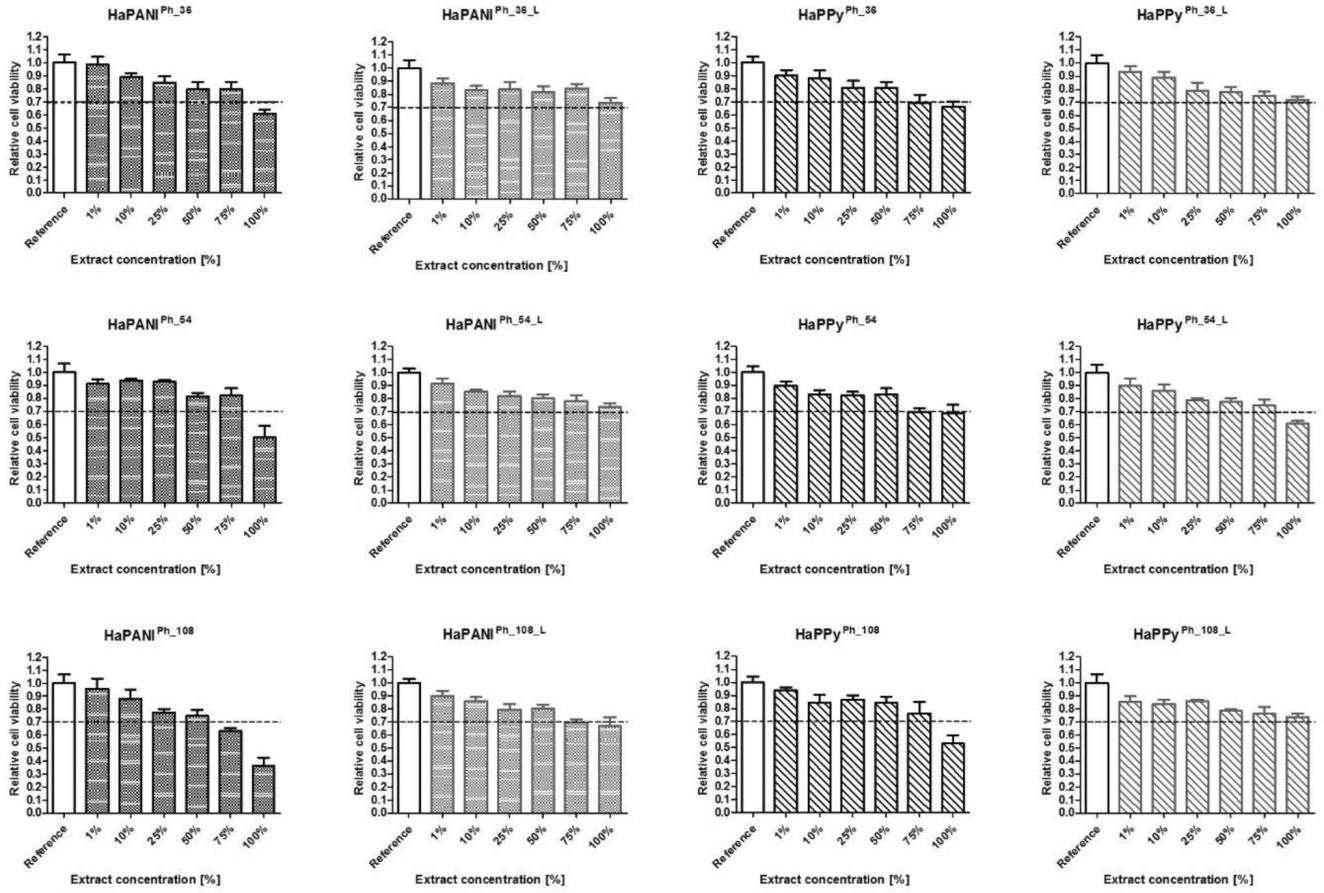
The cytotoxicity of physically crosslinked scaffolds. According to ISO standard 10993, a decrease in viability to below 70% of the reference corresponds to a cytotoxic effect on the part of the tested materials. Here the dashed line shows the threshold of cytotoxicity. All tests were conducted in quadruplicates within three repetitions. The average viability with its standard deviation are shown.

The behaviour of physically crosslinked samples (Fig. 8) prior to lyophilisation was similar to that observed for scaffolds crosslinked chemically, and only in a few cases did the samples show a cytotoxic effect, which was, moreover, observed at only the highest extract concentration. Only the highest extract concentration of native HaPANI^Ph^ scaffold significantly exceeded the cytotoxicity threshold, and the effect correlated with the concentration of HaPANI colloidal particles in the sample. Contrary to the situation observed for scaffolds prepared by chemical crosslinking, here lyophilisation decreased the negative impact of the scaffolds on cell viability. This could actually be observed in the case of all samples, especially in the case of HaPANI^Ph_L^, where none of the lyophilised hydrogel samples was cytotoxic.

The difference in the behaviours of lyophilized scaffolds prepared by chemical and physical crosslinking could, to some extent, be expected, as physically crosslinked scaffolds lack the possible residues of cytotoxic crosslinking agents, which can be released during the lyophlization process. We can also hypothesise that the difference is related to the different pore structures and the type of bound cytotoxic residual compounds within the scaffold structure. It seems that the CP-based colloids presented here do not substantially contribute to the overall cytotoxicity of the scaffolds, either in the case of HaPANI or HaPPy colloidal particles. Indeed, this is not surprising, as previous studies by Humpolicek et al. have shown that the cytotoxicities of both CPs are similar^40^.

### Cell adhesion, growth and ingrowth

**Figure 9:**
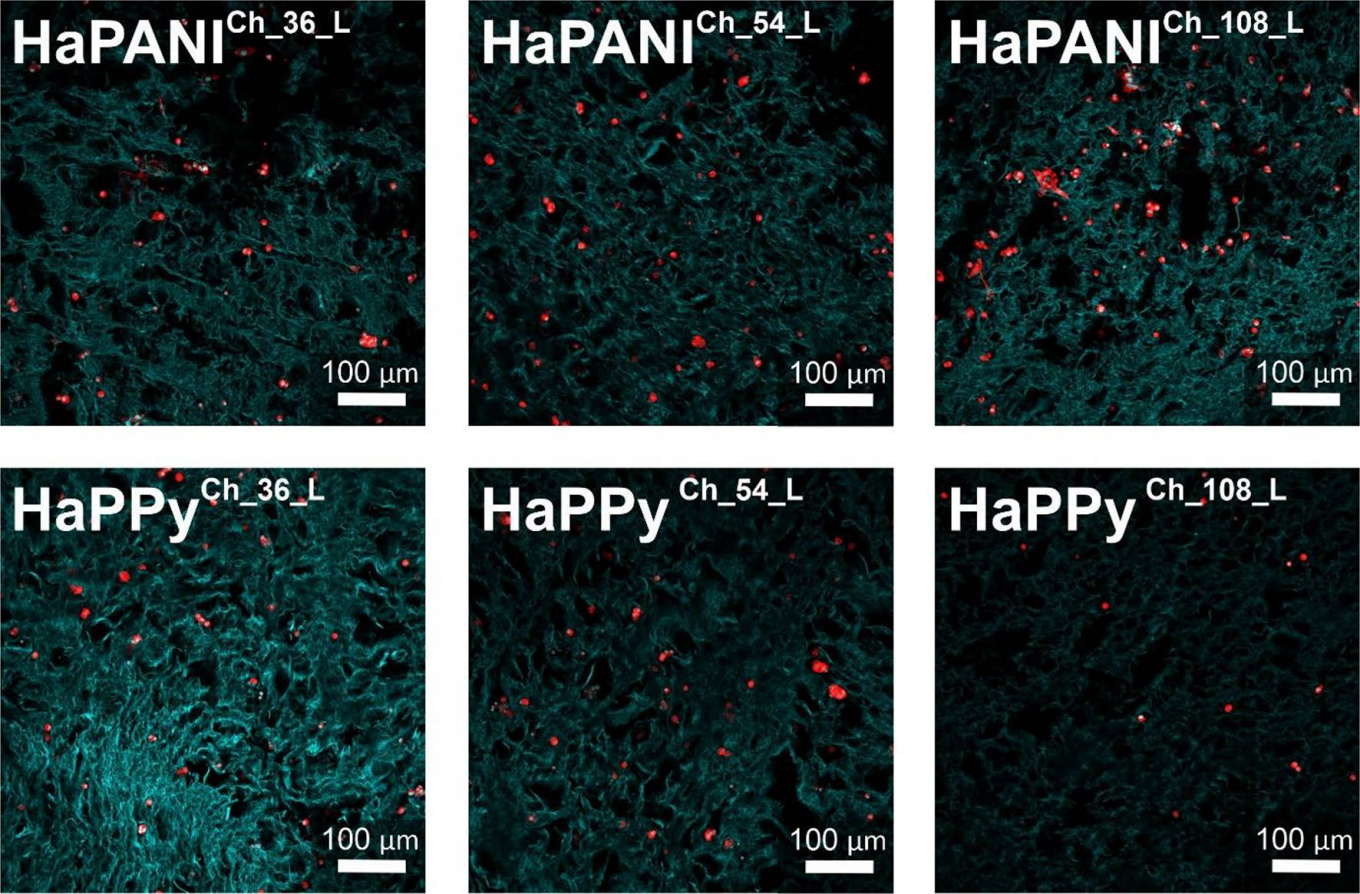
The adhesion and growth of cells on the surface of scaffolds. Cell nuclei were counterstained with Hoechst (blue); the cytoskeleton was counterstained with ActinRed (red). The green colour represents the hydrogel structure.

The cytotoxicity of scaffolds, which can be related to the composition of the material and the possible leaching of residual precursors or reagents under extraction, was low. The lyophilized samples were therefore subjected to another cytocompatibility study, this involving the determination of bio-interface properties represented by the ability of cells to adhere onto the scaffold surface and subsequently grow. The lyophilized samples were chosen, as their internal architecture was more appropriate for cell ingrowth than that of native scaffolds. Cells were seeded on the surfaces of scaffolds and cultivated for one week. The cells were able to adhere and grow on all surfaces. Representative pictures are presented in Fig. 9.

**Figure 10:**
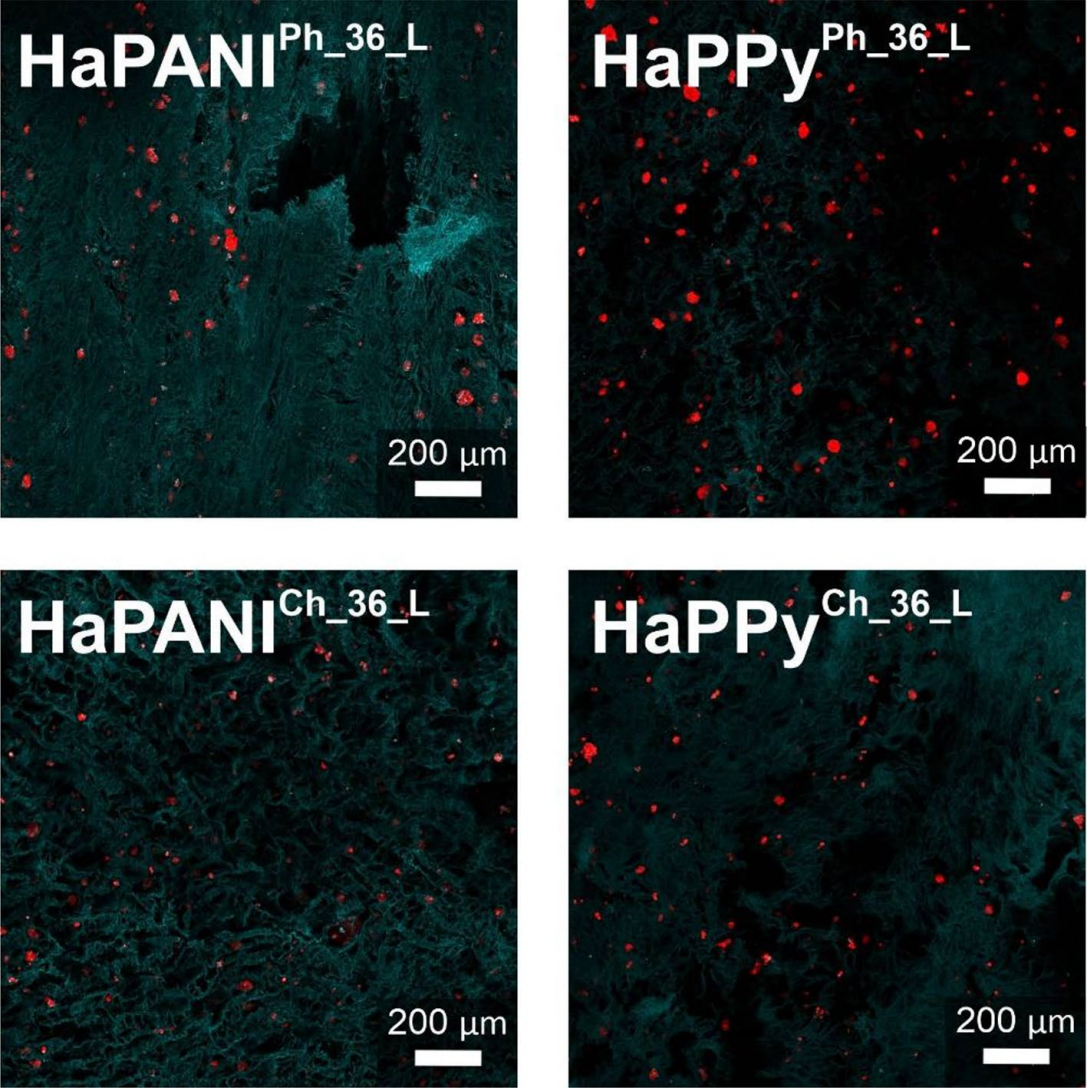
Cell ingrowth into the structure of scaffolds. Cell nuclei were counterstained with Hoechst (blue); the cytoskeleton was counterstained with actin red (red). The green colour represents the hydrogel structure. Cells were seeded onto the surface of scaffolds before cultivation in a bioreactor. The images were taken from the cross-sections of scaffolds, namely from the centre of sample and 0.5 cm below the surface. The images thus represent cells which grow into the structure of scaffolds from its surface.

Scaffolds suitable for tissue engineering must not only allow the attachment and growth of cells on their surfaces, but also be able to facilitate cell ingrowth into the structure of the scaffold. As mentioned above, an increasing concentration of HaPANI or HaPPy colloidal particles within the scaffold resulted in the higher cytotoxicity of the scaffold. The ingrowth of cells, allowed by their cultivation in a bioreactor mimicking in vivo conditions, was therefore tested only on samples with the lowest concentration (0.036%) of HaPANI and HaPPy (Fig. 10). Cells were seeded onto the surface of each scaffold and left to grow into the scaffold structure during cultivation in the bioreactor. Figure 10 show that cells were able to grow into the structure of the scaffold; thus, we can expect that only viable cells were counterstained there.

**Figure 11:**
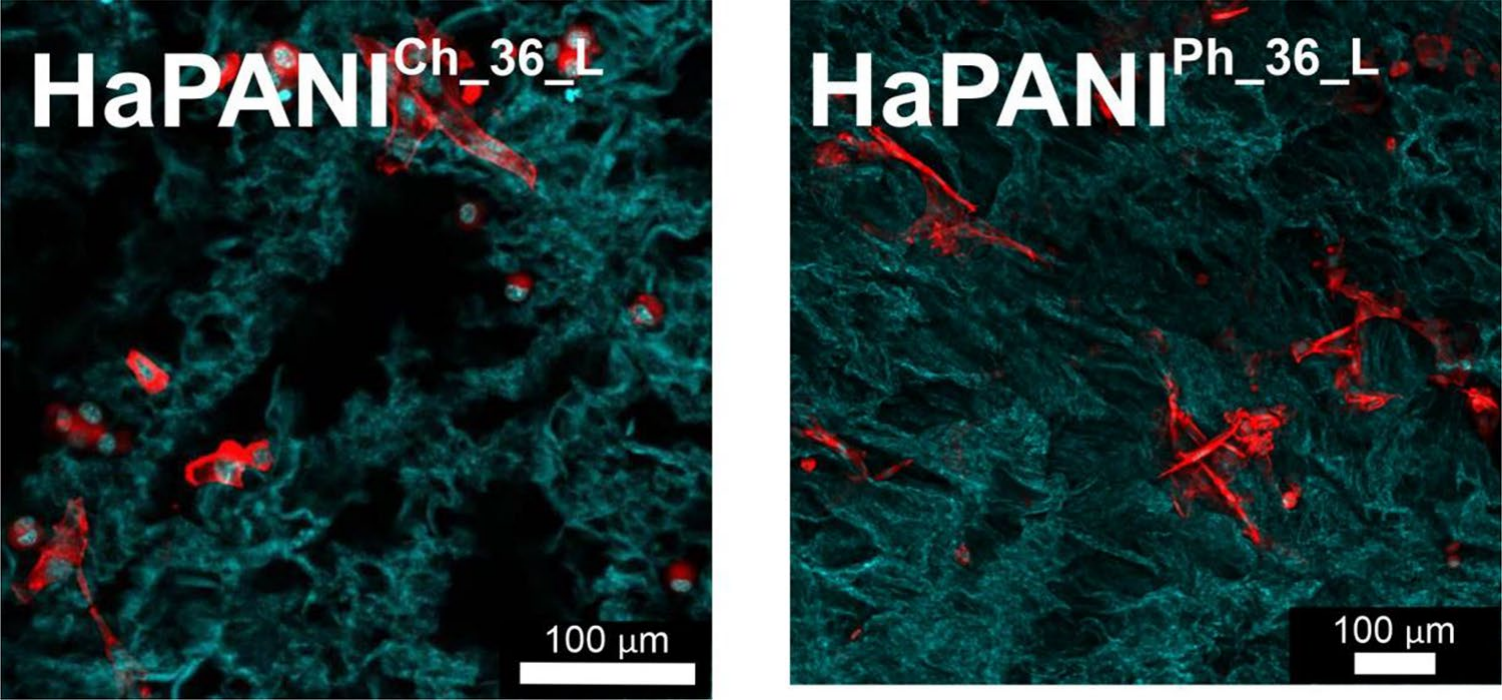
The morphology of cells within the structure of scaffolds. Cell nuclei were counterstained with Hoechst (blue); the cytoskeleton was counterstained with actin red (red). The green colour represents the hydrogel structure. Cells were seeded onto the surface of scaffolds before cultivation in a bioreactor. The images were taken from the cross-sections of scaffolds, namely from the centre of sample and 0.5 cm below the surface. The images thus represent cells which grow into the structure of scaffolds from its surface.

The counterstaining of cell cytoskeletons shows that cells with more round-like as well as with spindle-like structures were present (Fig. 11). These different cell shapes could be influenced by the scaffold architecture. The experiments with cell growth showed not only that the scaffolds prepared here were cytocompatible with respect to basic parameters such as the absence of cytotoxicity, but also that their bulk architecture created a friendly environment for cell ingrowth; thus, such scaffolds can be suitable for use in tissue engineering. Furthermore, their inherent conductivity, as a cell-instructive property, assured by the presence of CP-based colloids, opens up their potential use in a wide range of applications relating to the tissue engineering of electro-sensitive tissues.

## Conclusion

The preparation and composition of scaffolds have been widely studied for decades, as they are critical factors conditioning the application of scaffolds in tissue engineering. In addition, scaffolds should not only be passive supporting materials for cells, but also enable preferable responses to external stimuli leading to cell-instructiveness. One of the cell-instructive cues of materials is electrical conductivity, which is especially advantageous when electro-sensitive tissues, such as heart, neuronal, or muscle tissues, are considered. The incorporation of conductive polymers into the structures of scaffolds is an advantageous way of inducing conductivity in the materials. The poor solubility of CPs in an aqueous environment and their limited miscibility with other hydrophilic polymer matrices makes the fabrication of scaffolds based solely on CPs difficult. The novelty of the here-presented fabrication techniques lies in their removal of these shortcomings by preparing colloidal particles based on the conductive polymers PANI and PPy and incorporating them into the structure of the biocompatible and biodegradable polymer sodium hyaluronate. In our study, two crosslinking methods were employed—namely, chemical and physical—and the influence of composition and the route of crosslinking on the final material and the biological properties of the scaffolds were determined. According to the presented data, it can be concluded that both used crosslinking techniques allow the efficient incorporation of colloidal particles into the bulk polymer, thus making the final composites conductive. The characterization methods employed here revealed that the porosity, pore size, mechanical properties, and swelling of the studied samples were rather similar. Also, the results of cytotoxicity tests confirmed that the scaffolds were similarly cytocompatible with respect to this important characteristic. The effect of the lyophilization process on cytotoxicity was found to be greater than the crosslinking route used for preparation. Most crucially, tests conducted in bioreactors mimicking in vivo growth conditions showed that the scaffolds allow the adhesion and growth of cells on their surfaces; moreover, the cells were even able to grow into the structure of the scaffolds. The materials presented here thus represent electro-conductive and cytocompatible composites with potentially cell-instructive properties.

## Supplementary Information

Supplementary Information.
