# Supplementary material for: New approach to prepare cytocompatible 3D scaffolds via the combination of sodium hyaluronate and colloidal particles of conductive polymers
Source: Sci Rep. 2022 May 16;12:8065. doi: 10.1038/s41598-022-11678-8 (PMC9110748; doi:10.1038/s41598-022-11678-8)
Supplement: Supplementary file 1 — Supplementary Information. [file 41598_2022_11678_MOESM1_ESM.docx]

Supplementary material

New approach to the preparation of cytocompatible 3D scaffolds via the combination of sodium hyaluronate and colloidal particles of conductive polymers

Thanh Huong Truong^a^, Lenka Musilová^a,b^, Věra Kašpárková^a,b,*,^ Daniela Jasenská^a^, Petr Ponížil^b^, Antonín Minařík^b^, Eva Korábková^a^, Lukáš Münster^a^, Barbora Hanulíková ^a^, Aleš Mráček^a,b^, Petra Rejmontová^a^, Petr Humpolíček ^a,b*^

a Centre of Polymer Systems, Tomas Bata University in Zlin, Czech Republic

b Faculty of Technology, Tomas Bata University in Zlin, 760 01 Zlin, Czech Republic

Number of pages:

Number of figures: 4 (Figure S1-S4)

*
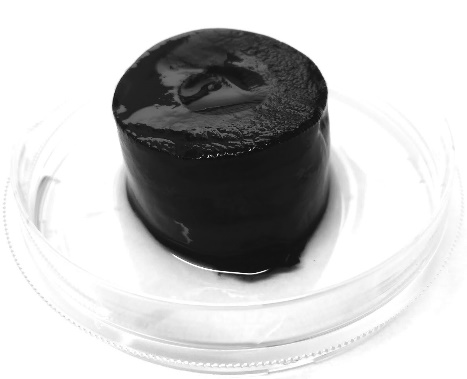
*

## *Figure S1. The sample used for testing of Young´s modulus (re: 2.4.2 Mechanical properties)*


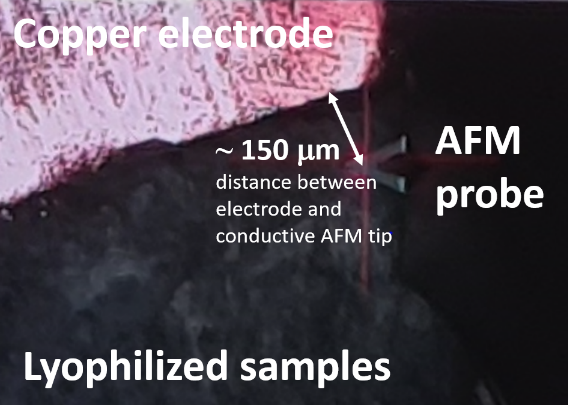


***Figure S2*** *Experimental setup for the characterization of surface electrical properties using the PeakForce TUNA AFM. Image from Dimension Icon digital camera. (re: 2.4.3 2.4.3.Surface topography and electrical properties)*

To ensure constant characterization conditions using PeakForce TUNA AFM, a special experimental setup was used. The copper tape with acrylic conductive adhesive, with resistivity 0.003 Ohm, was situated onto the polymer surface to a distance about 150 μm from the tip of the AFM probe.


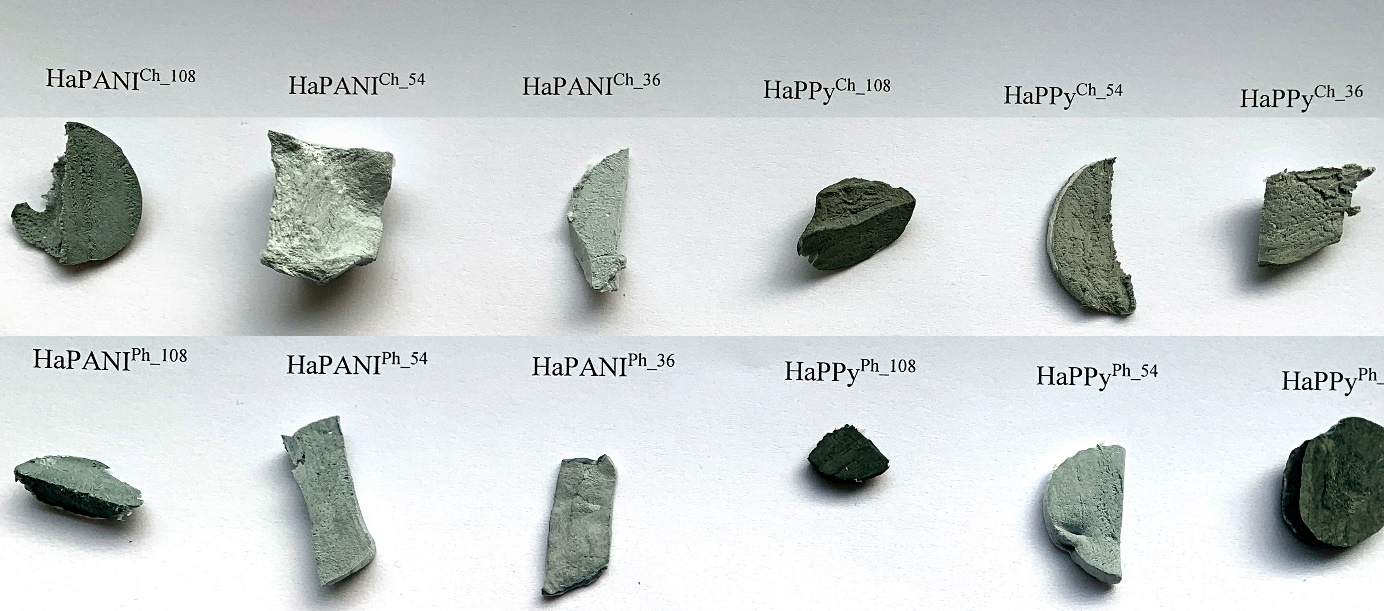


## *Figure S3 Macroscopic images of scaffolds (re: 2.4.4 and 2.4.5)*


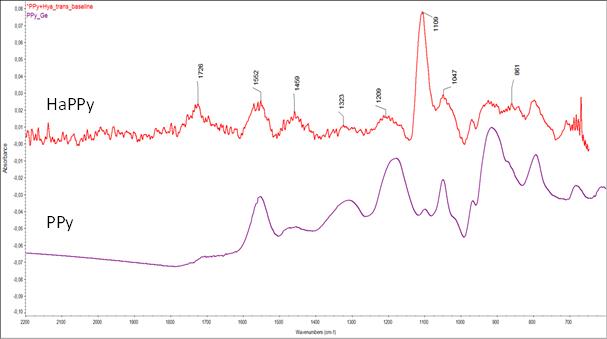


***Figure S4*** *Comparison of FTIR spectra recorded for pristine PPy and HaPPy composite.*

FTIT analysis was conducted by Nicolet 6700 (Thermo Scientific, USA). Spectra of pristine PPy were collected with ATR Ge crystal, HaPPy was analysed in transmission mode after applying colloid on silicone substrate. Both tests were run with 64 scans and a resolution of 4 cm^−1^. In the spectrum of HaPPy composite, characteristic bands of HA were detected: peak at 1726 cm^-1^ (C=O stretching vibration) and 1106 cm^-1^ (C-O stretching vibration).
